# Microplastic induces mitochondrial pathway mediated cellular apoptosis in mussel (*Mytilus galloprovincialis*) via inhibition of the AKT and ERK signaling pathway

**DOI:** 10.1038/s41420-023-01740-3

**Published:** 2023-12-06

**Authors:** Nhu Thi Quynh Mai, Ulziituya Batjargal, Won-Seok Kim, Ji-Hoon Kim, Ji-Won Park, Ihn-Sil Kwak, Byoung-San Moon

**Affiliations:** 1 Department of Medical Biotechnology, Yeungnam University, Gyeongsan, 38541 Korea; 2 Department of Integrative Biotechnology, Chonnam National University, Yeosu, 59626 Korea; 3 Department of Ocean Integrated Science, Chonnam National University, Yeosu, 59626 Korea

**Keywords:** Environmental biotechnology, Chemical ecology

## Abstract

Microplastics (MPs) is an escalating aquatic environmental crisis that poses significant threats to marine organisms, especially mussels. Here, we compare the cumulative toxic effects of the two most abundant morphotypes of MPs in the environment, microspheres, and microfibers, on the gill and digestive gland (DG) of *Mytilus galloprovincialis* in a dose-dependent (1, 10, and 100 mg/L) and time-dependent (1, 4, 7, 14, 21 days exposure) manner. DNA fragmentation assessment through TUNEL assay revealed consistency in the pattern of morphological disturbance degree and cell apoptosis proportions indicated by histopathological analysis. Upon the acute phase of exposure (day 1–4), gill and DG treated with low MPs concentration exhibited preserved morphology and low proportion of TUNEL+ cells. At higher concentrations, spherical and fibrous MP-induced structural impairments and DNA breakage occurred at distinct levels. 100 mg/L microfibers was lethal to all mussels on day 21, indicating the higher toxicity of the fibrous particles. During the chronic phase, both morphological abnormalities degree and DNA fragmentation level increased over time and with increasing concentration, but the differentials between the spherical and fibrous group was gradually reduced, particularly diminished in 10 and 100 mg/L in the last 2 weeks. Furthermore, analysis of transcriptional activities of key genes for apoptosis of 100 mg/L–day 14 groups revealed the upregulation of both intrinsic and extrinsic apoptotic induction pathway and increment in gene transcripts involving genotoxic stress and energy metabolism according to MP morphotypes. Overall, microfibers exert higher genotoxic effects on mussel. In response, mussels trigger more intense apoptotic responses together with enhanced energy metabolism to tolerate the adverse effects in a way related to the accumulation of stimuli.

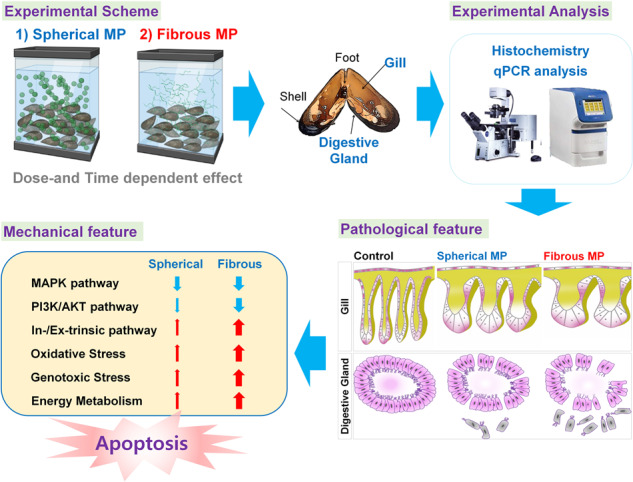

## Introduction

Microplastics (MPs) poses significant threats to ecosystems [1, 2], especially, organisms therein [3] due to high mobility and long residence times. Since MPs occupy the same size range as plankton, these particles are potentially up-taken by ingestion or by food-gathering structures of various marine organisms [4, 5]. Through the internalization into organisms with plankton-based diets, MPs not only affect their feeding activity and nutritional value, but also enter the food web, enlarging their influences on various species. In this respect, filter-feeders, such as mussels, appear to be the most vulnerable to this pollutant since the water-filtering activity and indiscriminate food intake exposes them to a large quantity of plastic particles [6, 7]. Indeed, MPs have been detected in various organs of mussels, causing morphological distortion and structure damage [3, 8–10]. In addition, cell apoptosis, increased oxidative stress and inflammation have been reported as consequences of MPs exposure [9, 11, 12]. In this respect, although several regulatory signaling pathways have been identified to trigger apoptosis initiation in bivalves [13–15], the mechanism governing the apoptotic death of cells in MPs contamination remains much to clarified. Also, whether different morphotypes of MPs results in different degree of apoptotic response, and how the acute and chronic effects of pollutants alter the degree of programmed cell death in mussels, have yet been demonstrated. Since understanding how mussels cope with this stress stimulus is crucial for intensifying the current knowledge of MP’s effects and developing effective strategies to mitigate the adverse effects of MP pollution, this study attempts to elaborate the difference in cytotoxicity caused by different MP types in mussel, focusing on *Mytilus galloprovincialis*.

This study emphasizes on the cumulative toxicity of two MP morphotypes, microsphere and microfibers, the two most abundant MPs morphotypes in the environment [16, 17], in *Mytilus galloprovincialis* by comparing their effects on histopathological alterations, DNA fragmentation in the gill and digestive gland (DG), which are the first and last organs of MPs exposure, in a dose- and time-dependent. We also devise a novel approach for assessing histopathological morphology in the gill that enables degree quantification. In parallel, transcriptional alterations in mussels on MPs exposure was also investigated, which suggested the differences in cytotoxic induction and cell responses mechanisms between MP morphotypes in mussels.

## Results

### Morphological disorders in mussel gill filaments induced by spherical and fibrous MPs

**Fig. 1:**
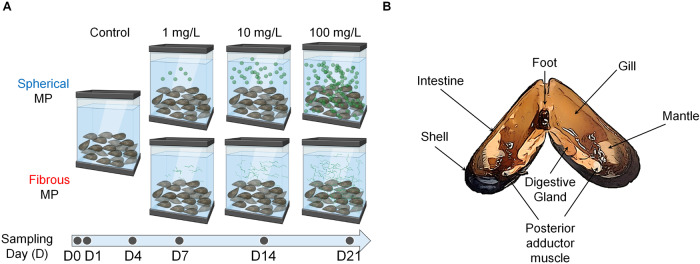
Dose- and time-dependent exposure of spherical and fibrous MPs on mussel. **A** Schematic showing the experimental design of the study. The collected mussels were allocated to glass aquaria (10 mussels each) and were exposed to nominal increasing concentrations of spherical, and fibrous MP for 21 days. Sampling was performed at each indicated timepoint. **B** Schematic depicting general anatomy of the mussel tissues in ventral view during the sampling process. In brief, the adductor muscles were first cut, and the valves were forced to open, hereby rupturing the connecting mantle parts.

**Fig. 2:**
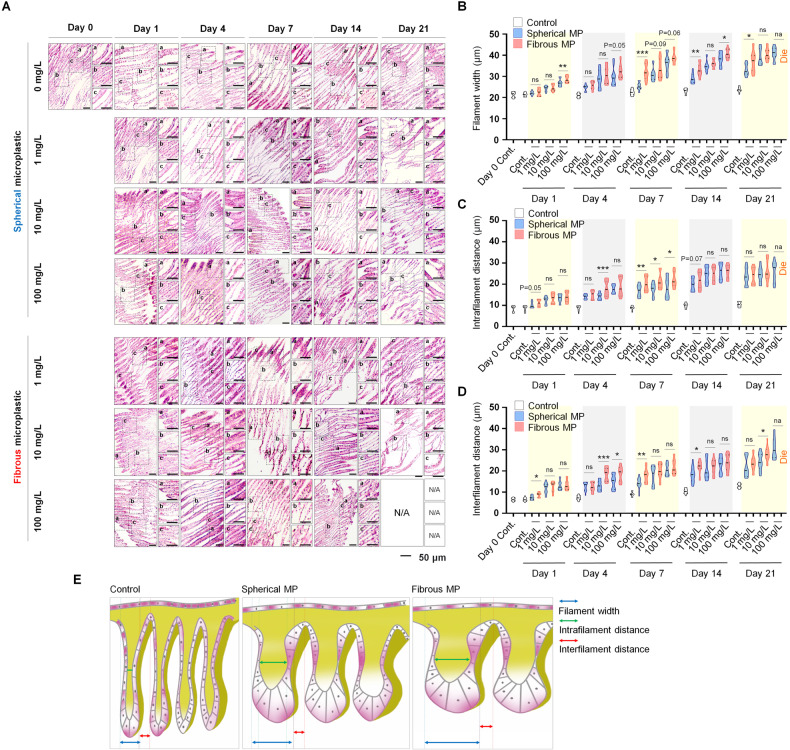
Histopathological assessment of gill filaments on exposure to different MP morphotypes over time. **A** H&E staining of the gills of each group. Scale bar: 50 µm. **B** Gill filament width of mussels in each group. **C** Intrafilamentous distance of mussel gill in each group. **D** Interfilamentous distance of mussel gill in each group. Data are presented as mean ± SD. One-way ANOVA with Turkey’s post hoc test was conducted to calculate significance (*p < 0.05, **p < 0.01, ***p < 0.001, *n* = 15). ns; not significant. **E** Schematic illustrating the effects of spherical and fibrous MP on mussel’s gill regarding pathological morphologies. Gill filament width was measure at 100 µm distant from the filament tip. Intrafilamentous distance was identified as the distance between two parallel epithelia tissues, measured at the intermediate zone of each gill filament. Interfilamentous distance was measured as the smallest distance between two parallel epithelia tissues. All measurements were determined by a line perpendicular to the gill lamella axis (parallel to most of the gill filaments in the figure).

To investigate the acute and chronic effects of MPs, we exposed mussels to increased concentrations (1, 10, 100 mg/L) of spherical and fibrous particles (Fig. 1A) for 21 days. The gill, the water-filtering organ with high risk of MPs exposure, and DG, the last organ subjecting to MPs from the indiscriminating ingestion (Fig. 1B), were taken into investigation. We first characterized MP’s toxicity on gill lamella by examining histopathological morphology. Through H&E staining, we observed the swelling in gill filaments and quantified this phenomenon by measuring gill filament width (Fig. 2A). Following low MP concentrations, gill filament exhibits preserved structure (~20 µm) in the first 4 days (Fig. 2B) then enlarged progressively with increasing concentrations. The highest width was recorded on day 21 (~45 µm), suggesting extensive hypertrophy. Notably, the sphere and fiber morphotypes demonstrated significant difference from day 1 at high concentration (100 mg/L) and keep showing distinct effects during the chronic phase (day 7–21). At the same time, we measured the intrafilamentous distance at their intermediate zone (Fig. 2C) and observed that epithelial cells composing gill filament became distant from one another following MPs treatment regardless of particle shape. Significant differences between MP concentrations were clearly shown on day 7, then became subtle during the later stages, indicating that intrafilamentous distance is not strongly affected by MP morphotypes. However, the progressive increases with dose and time emphasized MP’s effects on disorganization of the gill structure.

We next sought to find if the interfilamentous distance, identified as the smallest distance between two adjacent filaments, is altered under the effect of MPs. Interestingly, fibrous MP caused an initial increase in the inter-gill filament distance after 1-day exposure to MP (Fig. 2D). This parameter increased progressively with increased dose and prolonged exposure time, yet significant differences were not consistently recorded, revealing the huge variation in the distribution of gill filaments. Despite the discrepancy in interfilamentous distance within the same group, we observed that most samples at 1 mg/L exhibited well-preserved structure on day 1 with ciliated epithelia covering the external surface. Hypoplasia (loss of cilia) was observed with increased distance, especially from day 7 onwards, indicating MP-induced morphologic abnormalities. Until day 21, 100 mg/L microfiber was lethal to all the mussels, emphasizing its higher toxicity compared to microsphere. Except for this concentration, differences between the two morphotypes were scaled down during the chronic phase, indicating the cumulative effects caused by MP over the treating period. Our data, therefore, demonstrates that cytotoxicity and histopathology of MPs on the gill of mussel are synergistically affected by MP’s morphotype, concentration, and exposure time. Specifically, MPs lead to increased gill filament width, intra- and inter-filamentous distance in mussel gill, with higher kinetics in fibrous particles compared to spherical counterpart (Fig. 2E).

### Morphological disorders in mussel DG induced by spherical and fibrous MPs

**Fig. 3:**
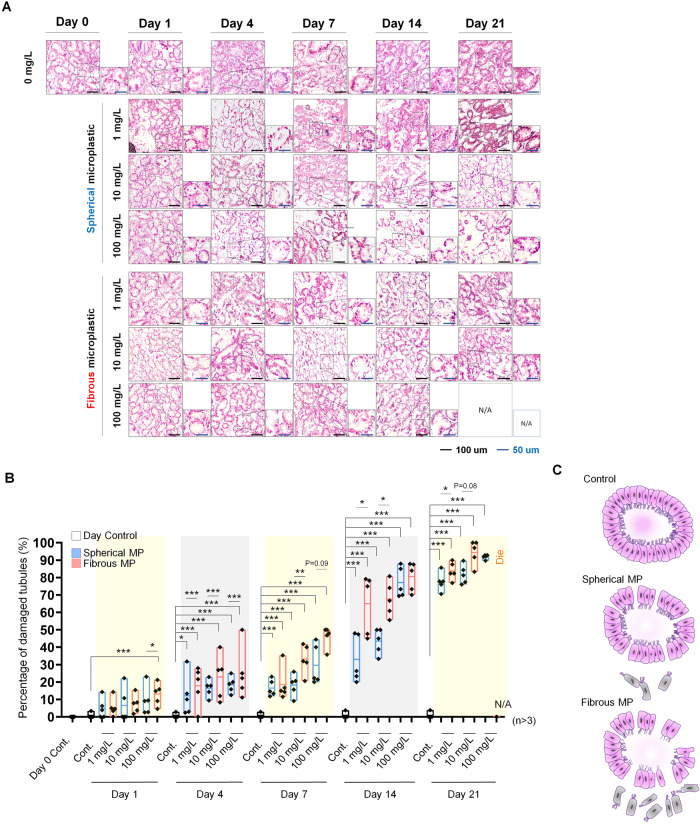
Histopathological assessment of digestive gland (DG) on exposure to different MP morphotypes over time. **A** H&E staining of the digestive tubules (DT) of each group. Scale bar: 50 µm. **B** Percentage of damaged DT in each group. Data are presented as mean ± SD. One-way ANOVA with Turkey’s post hoc test was conducted to calculate significance (*p < 0.05, **p < 0.01, ***p < 0.001, *n* = 5). **C** Schematic depicting the damage caused by MP morphotypes on mussel DT.

Prior studies have identified the accumulation of MP of mussel DG which leads to oxidative stress [18]. Therefore, we demonstrated the histopathological effects of spherical and fibrous MPs on DG via the percentage of injured digestive tubules (DT) observed in H&E staining (Fig. 3A, B). Indeed, early stages of MP treatment minorly altered the DT morphology when most samples showed normal structure with round/ovular shape with a columnar epithelium liner, with no or insignificant necrosis or damage of the tubule. Meanwhile, high concentration showed a significant increase in impairment proportion. This result reveals that 1-day exposure to 1 to 10 mg/L spherical or fiber MPs does not exert significant morphological alterations in DG. Notably, significant differences between the spherical and fibrous groups were observed at all concentrations on day 4, showing that DT is vulnerable to MP pollutant. In respect of the chronic phase, the principal histopathological conditions were distortions of the tubule structure, atrophy, and necrosis characterized by diffused nuclei and no clear distinction in some epithelial cells. Importantly, the prevalence of these structure disturbances increased significantly after 2 weeks, finally reaching a peak on day 21, when distortions were observed in almost all DTs. Additionally, the proportion of distorted DTs of each MP morphotype also exhibited clear dose-dependent tendencies from day 7 onwards, in which microfibers consistently induced more extensive abnormalities, thus, confirming that MP morphotype and exposure time synergized in promoting toxicity to mussel’s organs, and that fibrous MPs exert higher cytotoxic effects on mussel DGs.

### Exposure to spherical and fibrous MPs induces apoptotic death in mussel gill cells

**Fig. 4:**
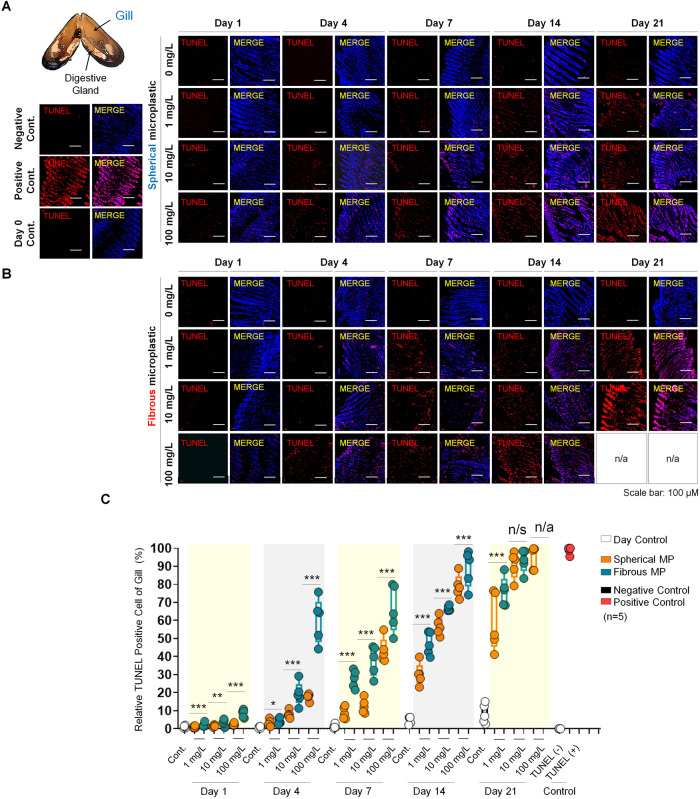
Cell apoptosis assessment of gill on exposure to different MP morphotypes over time. **A** TUNEL assay showing cells with DNA damage (red) of each group, counterstained with DAPI (blue) for cell nucleus. Scale bar, 100 µm. **B** Box plots displaying quantification for TUNEL+ cells in the gill of each group. One-way ANOVA with Turkey’s post hoc test was performed to calculate significance (*p < 0.05, **p < 0.01, ***p < 0.001, *n* = 5). n/s; not significant.

Since morphological assessment revealed the time- and dose-dependent histopathological disorders in the gill structure, we next examined whether MPs induce cell apoptosis through its hallmark, DNA damage, employing TUNEL assay. We observed that 1-day exposure to MPs resulted in low rates of TUNEL+ cells (below 10%) at all concentrations, regardless of pollutant’s morphotypes (Fig. 4A–C). On day 4, 1 mg/L spherical MP particles showed a marginal increment in the percentage of apoptotic cells (by ~4 times), while 10 mg/L and 100 mg/L led to substantial increases (nearly 10 and 22 times, respectively) compared to the control. Fibrous MPs observed a similar pattern of apoptosis percentages, but in a higher discrepancy among the three concentrations (by ~5, ~24, and ~75 times higher compared to the control). Notably, throughout these periods, the proportions of apoptotic gill cells in the fibrous groups were consistently higher than those with spheres exposure, especially at higher concentration, revealing that detrimental effects caused by MPs was correlated with the shape of MP particles. Regarding the chronic effects of MPs on mussel’s gill, our TUNEL data on day 7 showed a significant increased proportion of DNA damaged even at low concentration (1 mg/L). Notably, increasing MP concentration by 100 times (1 mg/L to 100 mg/L) leads to a 4.8- and 2.4-fold increase in the percentage of TUNEL+ gill cell of the sphere and fiber treated groups, indicating that dose significantly intensified apoptotic death of gill cells. This was supported by the data of day 14 and 21 which showed huge discrepancies in apoptotic proportion between different concentrations of the same MP morphotype. Fibrous MPs significantly enhanced apoptosis rate from the initial stage while spherical particles only showed minor effect, confirming higher cytotoxicity of microfibers. Interestingly, increased treatment period reduced the differences between microsphere and microfiber, suggesting that exposure time is positively correlated with MP cytotoxic effect. Together, both MP types induce apoptotic death of gill cells in either short or long-term exposure, but the acute effect exerted by microfibers is higher than that of microspheres.

### Exposure to spherical and fibrous MPs induces apoptotic death in mussel DG cells

**Fig. 5:**
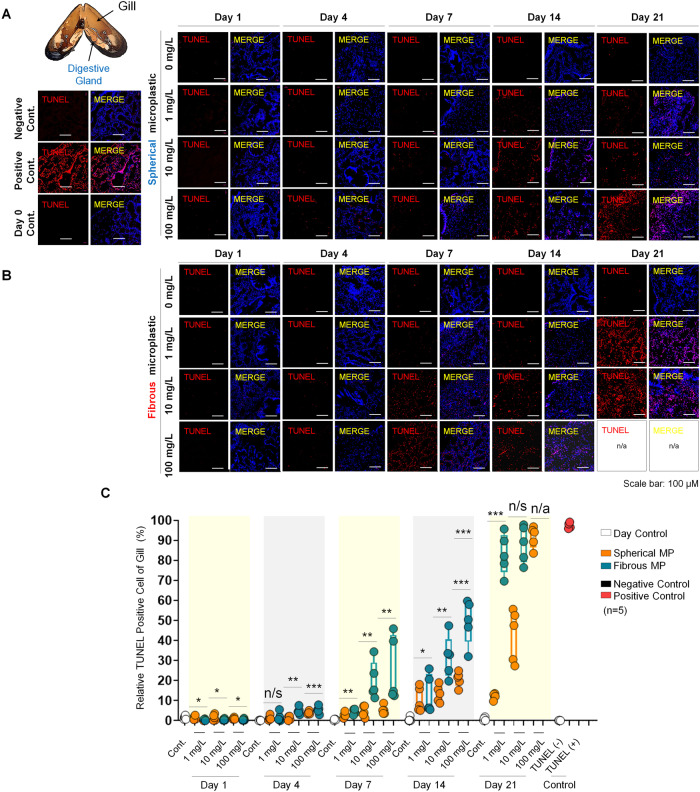
Cell apoptosis assessment of DG on exposure to different MP morphotypes over time. **A** TUNEL assay showing cells with DNA damage (red) of each group, counterstained with DAPI (blue) for cell nucleus. Scale bar, 100 µm. **B** Box plots displaying quantification for TUNEL+ cells in the gill of each group. One-way ANOVA with Turkey’s post hoc test was performed to calculate significance (*p < 0.05, **p < 0.01, ***p < 0.001, *n* = 5). n/s; not significant.

On the basis of histopathological analysis of the DG, we further examined the apoptotic effects of MPs on these cells. Conducting TUNEL assay, we observed that both MPs morphotypes induced DNA fragmentation in DG at a relatively low rate (below 10%) throughout the first 4 days (Fig. 5A–C). As MP dose increased, while day 1 showed no significant change in TUNEL+ cell proportion, day 4 demonstrated a gradually increased pattern in the fibrous groups and a minor increase in the spherical groups. Interestingly, although DG experience subtle changes during the acute phase compared to the gill, the level of apoptotic death caused by microfibers was significantly higher that what observed in the microspheres. By day 7, increased MP concentration promoted the apoptotic level by ~1.6 times and 1.9 times (spherical groups), and ~4.4 and ~5.6 folds (fibrous groups), respectively for 10 and 100 mg/L, compared to 1 mg/L. Furthermore, the DNA damage levels at 1 mg/L became considerable after 2 weeks and extending the treating period to day 14 and 21 showed similar pattern of apoptosis rate, indicating that MPs detrimental effect is positively correlated with concentration and exposure duration. 100 mg/L fibers became lethal dose to all mussels after 3 weeks, emphasizing the significance of exposure period to MP’s toxicity. Therefore, MP is an apoptotic causative factor in mussels DGs that is highly associated with particle types and treatment duration. The significantly higher apoptosis rate in the fibrous group compared to microspheres, which is consistent with what observed in the gill, emphasizes higher induction kinetics of microfibers on DNA fragmentation of mussel cells.

### Regulation of Bcl-2 and Bax, and inhibition of the PI3K/Akt and ERK pathway govern MP-induced apoptosis in mussel gill and DGs

**Fig. 6:**
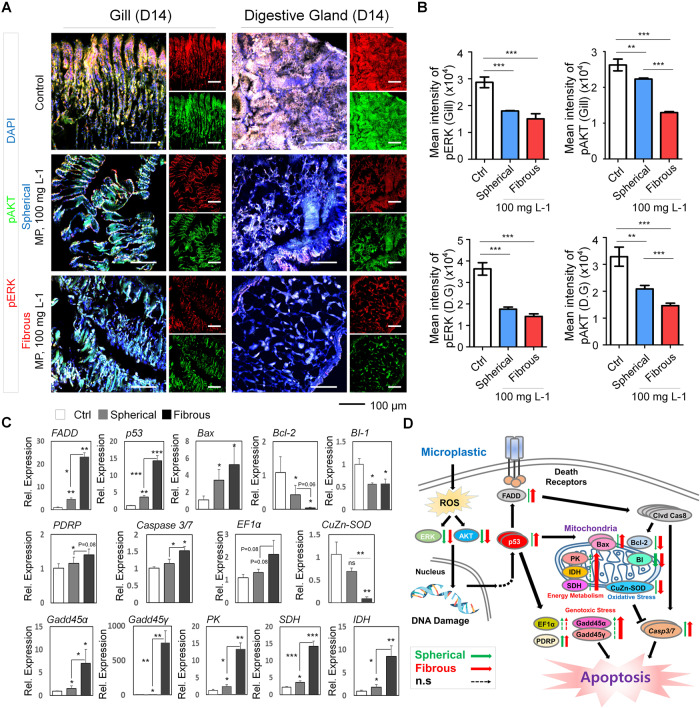
Alterations in gene expressions in the gill and DG of mussel under MP stressor. **A** Immunostaining for antibodies against DAPI (blue), pAKT (green), pERK (red) in the gill and DG of day 14–100 mg/L microsphere and microfiber groups. **B** Quantification for expression level of pERK and pAKT in gill and DG of each group. One-way ANOVA multiple comparison was performed to calculate significance (**p < 0.01, ***p < 0.001, *n* = 5). **C** Transcription level of target genes of the gill in day 14–100 mg/L microsphere and microfiber groups. Data are presented as mean ± SD. One-way ANOVA with Turkey’s post hoc test was conducted to calculate significance (*p < 0.05, **p < 0.01, ***p < 0.001, *n* = 3). n.s; not significant. **D** Schematic depicting the molecular pathways functioning in spherical and fibrous morphotypes MP-induced stress in mussels.

To elucidate the mechanism governing the apoptotic responses in the gill and DG cell to microsphere and fibers, we first visually investigated the expression level of pAKT and pERK, the two key proteins involved in two crucial pathways for cell survival (phosphatidylinositol 3-kinase (PI3K)/AKT and ERK pathway, respectively) targeting the highest treatment dose and duration that showed the highest toxicity. However, since all mussels exposed to 100 mg/L fibrous MP had died on day 21, the 100 mg/L–day 14 groups were chosen for immunohistochemistry analysis (Fig. 6A, B). Compared to the control, pERK and pAKT protein levels was significantly reduced in both gill and DG of the treated groups. Interestingly, coinciding with the higher cytotoxicity, we observed a lower level of pAKT in the gill and DG of the fibrous group. The suppression on the pERK and pAKT expression levels suggested the deregulation of ERK and Akt pathway in gill and DGs in response to MP exposure. We then performed RT-qPCR of major genes functioning in apoptosis process (Fig. 6C). Notably, we found upregulations in the transcription of genes encoding FADD (Fas-associated protein with death domain) which is crucial for the extrinsic apoptotic pathway. In regard to the intrinsic apoptotic pathway, we investigated the RNA level of p53 (tumor protein p53), a cellular stress biomarker in mussel [19–21], PDRP, BI-1 (Bax-inhibitor 1), and Bcl2 family representative members, including Bcl2, and Bax (Bcl-2 Associated X-protein) [22], and found significant increases except for BI-1 and Bcl2 which showed a reverse tendency. Specifically, following 14-day exposure to MPs, transcription level of p53 and Bax augmented by 3 and 1.6 times, respectively, in the fibrous compared to the spherical group, suggesting higher apoptotic level. Additionally, Caspase 3/7, which functions in both apoptotic pathways, showed significant upregulations in the microfiber group compared to the microsphere counterpart. These transcriptional upregulations, which were remarkably enhanced in the fibrous group, revealed the activation of both apoptotic pathways in mussels following MP exposure. At the same time, we found minor increases in the transcription rate of EF-1α (elongation factor 1α) which regulates protein synthesis and involves in cell apoptosis, suggesting that this gene might not engage in mussel cell’s apoptotic response to MP pollution. To further characterize the effect of MPs on mussel gill and DG, we targeted genes associated with pollutant-induced stress. We found a drastic reduction in transcripts of CuZn-SOD (zinc copper superoxide dismutase) gene, an antioxidant defense biomarker in the fibrous group compared to that of the microspheres, supporting the significantly increased apoptosis rate in this group. Interestingly, our RT-qPCR analysis detected substantial increment in the transcripts level of Gadd45α, Gadd45γ (growth arrest and DNA damage inducible protein) in treated groups compared to the control, reflecting genotoxic stress. Significantly, the upregulation in microfibers was doubled and by far higher in Gadd45α, and Gadd45γ, respectively, compared to that of microspheres, confirming the intense toxicity of fibrous particles. Interestingly, *PK* (pyruvate kinase), *SDH* (succinate dehydrogenase), and *IDH* (isocitrate dehydrogenase) genes were also differentially upregulated with remarkably higher levels in the fibrous group compared to the spherical, suggesting increased energy metabolism. Based on these findings, we devised the mechanism of DNA fragmentation induction in mussel in response to MP pollution regulated by extrinsic and intrinsic apoptotic pathways (Fig. 6D). Taken together, our data demonstrated that MP contamination significantly aggravates apoptosis and activates genes involving genotoxic stress that leads to promoted mitochondria respiration of host to fulfils the increased demand of ATP.

## Discussion

The ubiquitous dispersion of MPs, specifically in the aquatic environment, is a considerable issue due to its significant threat to marine ecosystems, and potentially to human health, by entering the marine food-web [7, 23–25]. Therefore, investigation of the accumulation, ultimate fate and quantification of their toxicity in marine organisms, are crucially important for understanding of the risks and mitigating this globally significant issue. Here, we aimed to compare the toxicity kinetics of the two most prevailing morphotypes of MPs in real environment, sphere and fiber, focusing on the first and last organs exposing to MPs. In the literature, cytotoxicity of various forms of MPs have been investigated, considering the ingestion, accumulation and egestion in bivalves, reflecting microbeads elimination out of the intestine [26]. Notwithstanding, a recent study has pointed out that beyond ingestion, MPs are preferentially adhere to tissue surface, hence, potentially prolong their maintenance in organism’s body [27]. Given that the depuration of plastic particles is incomplete, with 10% of the internalized MPs remaining inside the mussels [8], the toxicity of MPs might be stronger when egestion is excluded from the investigation. For that reason, MP morphotype, treatment concentration and exposure duration were taken into investigation in our study without considering the impact of egestion, to characterize the chronic and acute effects of MPs and compare the degree of damage in the gill and DG in *Mytilus galloprovincialis*.

At the morphological level, our H&E staining demonstrated distortion in the gill and DG structure in a dose and time-dependent manner. Specifically, in accordant with a prior study, we observed hypertrophy, deciliation [9], and disorganization of the gills epithelium [9, 28, 29] as prominent structure disorders in the gill, and atrophy, and disrupted DTs as major abnormal morphology in mussel DG. Notably, unlike previous study, which focused on the percentage of cells carrying morphological distortion, here, to reflect the degree of histopathological alterations, we assessed for the first time the gill filament width together with intra-, and inter-gill filaments distance. The increment in gill filament width over time and increasing treatment dose reflected the promoted hypertrophy and epithelial thickening as the toxicity of MPs enhances. In addition, the expansion in intra-gill filamentous distance could serve as an indicator for the gill vacuolization phenomenon, which was previously reported as an effect of MPs in marine mussels [30]. Furthermore, the distance between gill filaments, which showed a noticeable inflated trend relative to increased dose and time, displays the degree of disorganization of mussel epithelium in correlation with MP toxicity. Assessment of detrimental effects with these approaches, therefore, allows the demonstration of abnormality degrees, enables the reflection of histopathological alterations progress, thereby facilitating toxicity investigation. Concomitantly, our histological evidence was supported by the assessment of DNA fragmentation, an event leads to apoptotic death as a cell response. The apoptosis proportion accorded with morphological distortion rate, proving the higher toxicity kinetics of the fibrous morphotype compared to microspheres, and emphasized the positive correlations between toxicity and exposure time. Extending the treatment mitigates the pace in the effects of spherical and fibrous groups, implicating the accumulation of MP’s toxicity over time.

Beyond the physiological and apoptotic response, MPs exposure has been shown to trigger transcriptomic alternations [30, 31], especially in the genes involving in oxidative stress, apoptosis, and immune activity. First, we showed the reduction of pERK and pAKT pathway in gill and DG of the D14–100 mg/L group through immunostaining, based on a prior study which performed immunoblot on mussel using human antibodies [32]. Further investigating apoptosis responses through RT-qPCR analysis provided additional insights on molecular mechanisms modulated by MPs in the gill of mussels. Until now, apoptosis is known to be mediated by two major pathways, the extrinsic or death receptor pathway, and the intrinsic or mitochondrial pathway, both of which involves the activation of caspase [33]. In respect of the mitochondrial apoptosis, we observed significant reduction in the transcription level of antiapoptotic gene Bcl-2 in parallel with augmentation in transcription activity of Bax which trigger permeabilization of the mitochondrial outer membrane (MOM), the hallmark of the intrinsic apoptosis branch. The occurrence of this hallmark event subsequently triggers the release of cytochrome c into cytoplasm, facilitating the formation of apoptosome complex that in turn, induces executioner caspase proteins [34] that mediates DNA fragmentation [35, 36]. We also observed that BI-1, which inhibits Bax activation to protect the cells against ER-stress induced apoptosis [37], was downregulated, supporting the exaggerated apoptotic death in the MP exposed groups. The enhanced transcription level of Caspase 3/7 recorded in the treatment groups, supports this notion. At the same time, MPs also triggered the extrinsic pathway through direct activation of adapter protein gene FADD that was differentially upregulated after MP exposure, especially in the fibrous group, consequently triggers the executioner phase of apoptosis [33]. Furthermore, the lower transcriptional activities of CuZn-SOD suggested the perturbations in antioxidant defense. Furthermore, the onset of DNA damage could be due to genotoxic stress, since Gadd45α and Gadd45γ, which functions in cell cycle checkpoint [38], DNA repair and cellular response to DNA-damaging agents [19, 39], was found to be upregulated. This rapid activation of cellular stress responses is also mediated by p53 which functions as tetrameric transcription factor, as suggested in previous study [40]. Consistently, our result showed the increment in p53 transcripts level, supporting the induction of cellular stress under MP exposure. Interestingly, we also found the association of MP-induced oxidative stress to alterations in mussel’s metabolism. PK, IDH, and SDH showed a drastic increment in transcription level, revealing the rapid activation of glycolysis and Kreb cycles, marking an increased energy metabolism demand to compensate the implementation of antioxidant and detoxification processes, along with DNA repair mechanisms, thereby, enabling the tolerance and homeostasis maintenance of mussels with the adverse environment [41]. This notion is consistent with a prior research, which showed a 25% increase in energy consumption of mussels experiencing a 14-day treatment with high concentration polystyrene microspheres [42]. Additionally, the indiscriminating intake of MPs could also result in promoted mechanical digestion, which increases the organism’s energy consumption, as suggested in a previous study [43]. Despite these findings, EF-1α transcripts level exhibited increasing pattern over spherical and fibrous groups, but no significant difference was detected. In a prior study, EF-1α was employed as the reference gene to normalize the expression level of investigated candidates [44]. Since this protein has been indicated to function in expediting protein synthesis, regulating cytoskeleton and apoptotic program [45, 46] in other cell lines but yet well-characterized in bivalves, whether protein synthesis is altered under MP-induced stress awaits further investigations.

In conclusion, our study devises a novel approach to assess the degree of histopathological alternations in mussel gill in toxicology study. We showed that microfibers exerted higher toxic effects on mussels compared to microspheres. Chronic exposure with increasing concentration also exaggerated the level of morphological abnormalities, DNA fragmentation, and transcriptional activities modification. In addition to oxidative status and apoptosis response, MPs also induce alterations in metabolism, and repair response, in ways related to the accumulation of pollutant-induced stress.

## Materials and methods

### Maintenance of mussels and microplastic exposure

About 210 mussels *Mytilus galloprovincialis* (>5 cm in shell length) purchased from Yeosu Fish Market of Korea were washed with seawater then acclimatized for 7 days under laboratory conditions. Mussels were randomly allocated to three sets, each including 7 glass aquariums containing 2 L sand-filtered seawater. Each aquarium reared 10 live mussels that were fed every day with microalgae using a 2:1 ratio mixture of Shellfish Diet 1800 (Reed Mariculture Inc., Campbell, CA, USA) and Nanno 3600 (Reed Mariculture Inc.), prepared following manufacturer’s instruction. Water quality was checked regularly (salinity 25 ± 1‰, dissolved O_2_ > 60%, pH 7.5 ± 0.5, temperature 19 ± 0.5 °C). Spherical polyethylene (PE) (27–32 μm, density 1 g/cm^3^, Cospheric, CA, United States) and fibrous polyethylene terephthalate (PET) (200–400 μm, density 1.4 g/cm^3^, Korea Institute of Industrial Technology, Korea) were used for the exposure.

### Sampling

Mussels were sampled on day 0, 1, 4, 7, 14, and 21. At each stage, three mussels were taken from each tank and undergone tissue isolation. Tissues were washed in PBS, then fixed overnight in 4% (v/v) paraformaldehyde (PFA) at 4 °C prior to paraffinization for histology analyses, or stored at −80 °C until RNA extraction.

### Paraffinization, deparaffinization and rehydration

The fixed tissues underwent paraffin block using tissue processor and paraffin embedding machine (Leica, IL, USA). 4-μm sections were generated with rotary microtome (Roundfin RD-315, Shenyang, China) and transferred onto glass slides. Prior to staining, deparaffinization and hydration was performed using series of Xylene (3 changes), Xylene: 100% Ethanol (1:1 ratio, v/v), 100%, 95%, 80% Ethanol, and DW (10 min per change).

### TUNEL assay

TUNEL assay for DNA fragment detection was performed using One-step TUNEL In Situ Apoptosis Kit (Elabscience, Texas, USA) following the manufacturer’s instructions.

### H&E staining

Rehydrated samples were stained in Hematoxylin (5 min), followed by a 10-min rinse under tap water, and Eosin stain (2 min). After rinsing under tap water (10 min), dehydration using series of step-by-step ethanol (70%, 80%, 95%, and 100%), followed by Xylene (3 changes, 5 min per change) was performed. The slides were mounted with aqueous mounting solution (DAKO, Carpinteria, CA, USA) and subjected to imaging.

### Immunohistochemistry staining

Following rehydration, antigen retriever process was performed by boiling the slides in citrate buffer (10 mM Sodium citrate, 0.05% Tween 20, pH 6.0) for 20 min. Samples were blocked in 5% BSA for 30 min, then incubated overnight at 4 °C with primary antibodies: rabbit anti-AKT (phospho S473, Abcam Inc., ab81283), mouse anti-Erk1/2 (Phospho-p44/42 MAPK Thr202/Tyr204, Cell Signaling Technology, Inc., #9106). After three PBS washes (10 min each), the slides were incubated with anti-mouse Alexa Fluor 555-conjugated IgG (Abcam Inc., ab150114), anti-rabbit Alexa Fluor 488-conjugated IgG (Abcam Inc., ab150077) in 1% BSA for 1 h at RT and counterstained with 4′,6-diamidino-2-phenylindole (DAPI). Images were obtained using confocal microscopy (K1 Fluo, Nanoscope Systems lnc., Korea). All images are representative of samples obtained from at least three separate experiments.

### RNA extraction and RT-qPCR

Total RNA from the gills was extracted using TRIzol reagent (Invitrogen, CA, USA) according to the manufacturer’s instructions. cDNA was synthesized using iScript Reverse Transcription Supermix (1708841, Bio-Rad, CA, USA). qPCR was carried out with iTaq Universal SYBR Green Master Mix (Bio-Rad). The PCR program started with UDG activation at 50 °C for 2 min, Dual-Lock DNA polymerase at 95 °C for 2 min, followed by 40 cycles of denaturation at 95 °C for 15 s, annealing at 60 °C for 15 s, extension at 72 °C for 1 min. Triplicate reactions were carried out for each sample. The relative expression levels of each target gene were obtained by normalizing its CT-values with those of *GAPDH*. Primer sets are listed in Table S1.

### Statistical analysis

All statistical analysis was performed using GraphPad Prism 9 software. Quantification on H&E staining was conducted using ImageJ Software. Data were checked for normality using Shapiro-Wilk test, *p* < 0.05. ANOVA followed by Tukey’s post hoc tests were performed to compare the difference between MP concentrations and exposure time. For RT-qPCR data, one-way ANOVA (Bonferroni’s Multiple Comparison test) was performed to compare the investigated groups. Data obtained from at least three independent experiments (*n* = 3) were averaged and represented as means ± SD as stated in the Figure Legends. In all cases, the *p*-value less than 0.05 was considered significant: *p < 0.05, **p < 0.01, ***p < 0.001.

### Supplementary information

Table S1

## Data Availability

All data are available in the main text.
