# Supplementary material for: Microplastic induces mitochondrial pathway mediated cellular apoptosis in mussel (Mytilus galloprovincialis) via inhibition of the AKT and ERK signaling pathway
Source: Cell Death Discov. 2023 Dec 6;9:442. doi: 10.1038/s41420-023-01740-3 (PMC10700607; doi:10.1038/s41420-023-01740-3)
Supplement: Supplementary file 1 — Table S1 [file 41420_2023_1740_MOESM1_ESM.docx]

**Microplastic induces mitochondrial pathway mediated cellular apoptosis in mussel (*Mytilus galloprovincialis*) via inhibition of the AKT and ERK signaling pathway**

Nhu Thi Quynh Mai^1,2^, Ulziituya Batjargal^2^, Won-Seok Kim^3^, Ji-Hoon Kim^3^, Ji-Won Park^3^, Ihn-Sil Kwak ^3, *^, Byoung-San Moon^1,2, *^

^1^ Department of Medical Biotechnology, Yeungnam University, Gyeongsan 38541, Korea.

^2^ Department of Integrative Biotechnology, Chonnam National University, Yeosu, 59626, Korea.

^3^ Department of Ocean Integrated Science, Chonnam National University, Yeosu 59626, Korea

^*^ Email: inkwak@hotmail.com, bsmoon@yu.ac.kr

Table S1

Table S1: Sequences of primers used for the RT-qPCR assay.

| Accession no. | Transcript | Primer sequences (5’-3’) | Referen-usece | |
| --- | --- | --- | --- | --- |
| AB103129 | **28S rRNA** | **F: AAGCGAGAAAAGAAACTAAC**  **R: TTTACCTCTAAGCGGTTTCAC** | (1) |  |
| AF157491 | **β-actin** | **F: GCACTTCCTCACGCTATCGTC**  **R: TGTCCATCTGGCAGTTCGTAGC** | (2) | |
| AJ623737 | **Gadd45-γ** | **F: TCTGTTTCGGCCATCTCTGGT**  **R: GCACAGGAAGACGGCAGAATT** | (3) | |
| AJ623737 | **Gadd45-α** | **F: AAGAAATGTGAACAACATAGGGTTTGC**  **R: ACAACAATTCTGCCGTCTTCCT** | (4) | |
| AB162021 | **EF-1α** | **F: CTGAGATGGGAAAAGGCTCCTT**  **R: GACAAACTGAAGGCTGAGCG** |  |  |
| DQ158079 | **p53** | **F: CAACAACTTGCCCAATCCGATTTAA**  **R: GGTTCTTGGACATGTTCAGGTTTCA** | (5) | |
| FM177867 | **Cu/Zn-SOD** | **F: TCGCTTTCAGTCAACAGAATGG**  **R: CCAAACTCGTGAACGTGGAA** |  |  |
| HQ424453 | **Casp3/7** | **F: AGGCCCAGGTACAATATCCA**  **R: ATCGCTTCCTTTACGGTCTG** |  |  |
| KF051276.1 | **FADD** | **F: GGGAGACATGGAATTTAACTCG**  **R: GCAGTTTCCACTTTTTCCATG** |  |  |
| AM076953 | **PK** | **F: CTCAGTGTCAGGAAGGCTTG**  **R: CCACAGAATCACATGCTGGA** |  |  |
| JQ429386.1 | **IDH** | **F: CCTCTGGTGTTCAGCAGATC**  **R: ACCCCACTAGCACTGAAGAT** |  |  |
| XM_011434596 | **SHD (NADP-dependent)** | **F: GGTGGAGCAGGATTAAGAGC**  **R: CCATGTGACCTAAAGCTGCA** |  |  |
| KC545830 | **Bax** | **F: CCAACAGGTCCACCATTAGAAC**  **R: CTCTTGGCCACAGTTAGGAATG** | (6) | |
| KC545829 | **Bcl-2** | **F: AGATAACGGTGGTTGGCAAG**  **R: TAACGCCATTGCGCCTAT** |  |  |
| KC545831 | **BI-1** | **F: GGCCAGTTTTCTCACCTCCT**  **R: CCAATCCATGACTGGACCAA** |  |  |
| KC545828 | **PDRP** | **F: CTGCCAAAGAAAGCTACAAAGAAG**  **R: CCTTTGACAATGGATTGAGGTT** |  |  |
| F, Forward; R, reverse | | | | |

**REFERENCES**:

1. Cellura C, Toubiana M, Parrinello N, Roch P. Specific expression of antimicrobial peptide and HSP70 genes in response to heat-shock and several bacterial challenges in mussels. Fish & Shellfish Immunology. 2007;22(4):340–50.

2. Gourgou E, Aggeli IK, Beis I, Gaitanaki C. Hyperthermia-induced Hsp70 and MT20 transcriptional upregulation are mediated by p38-MAPK and JNKs in Mytilus galloprovincialis (Lamarck); a pro-survival response. Journal of Experimental Biology. 2010;213(2):347–57.

3. Varotto L, Domeneghetti S, Rosani U, Manfrin C, Cajaraville MP, Raccanelli S, et al. DNA Damage and Transcriptional Changes in the Gills of Mytilus galloprovincialis Exposed to Nanomolar Doses of Combined Metal Salts (Cd, Cu, Hg). PLOS ONE. 2013;8(1):e54602.

4. Ruiz P, Katsumiti A, Nieto JA, Bori J, Jimeno-Romero A, Reip P, et al. Short-term effects on antioxidant enzymes and long-term genotoxic and carcinogenic potential of CuO nanoparticles compared to bulk CuO and ionic copper in mussels Mytilus galloprovincialis. Marine environmental research. 2015;111:107–20.

5. Détrée C, Gallardo-Escárate C. Polyethylene microbeads induce transcriptional responses with tissue-dependent patterns in the mussel Mytilus galloprovincialis. Journal of Molluscan Studies. 2017;83(2):220–5.

6. Estevez-Calvar N, Romero A, Figueras A, Novoa B. Genes of the mitochondrial apoptotic pathway in Mytilus galloprovincialis. PloS one. 2013;8(4):e61502.
